# Four Severity Levels for Grading the Tortuosity of a Retinal Fundus Image

**DOI:** 10.3390/jimaging8100258

**Published:** 2022-09-22

**Authors:** Sufian Abdul Qader Badawi, Maen Takruri, Yaman Albadawi, Muazzam A. Khan Khattak, Ajay Kamath Nileshwar, Emad Mosalam

**Affiliations:** 1 Department of Computing, School of Electrical Engineering and Computer Science, National University of Sciences and Technology (NUST), Islamabad 44000, Pakistan; 2 Center for Information, Communication and Networking Education and Innovation (ICONET), American University of Ras Al Khaimah, Ras Al Khaimah 72603, United Arab Emirates; 3 Department of Computer Science and Engineering, College of Engineering, American University of Sharjah, Sharjah 26666, United Arab Emirates; 4 Department of Computer Science, Quaid-i-Azam University, Islamabad 45320, Pakistan; 5 Department of Ophthalmology, RAK College of Medical Sciences, Ras Al Khaimah Campus, RAK Medical and Health Sciences University, Ras Al Khaimah 11172, United Arab Emirates; 6 Saqr Hospital, Emirates Health Services, Ras Al Khaimah P.O. Box 5450, United Arab Emirates; 7 Dr. Emad Mosalam Eye Clinic, Ras Al Khaimah P.O. Box 5450, United Arab Emirates

**Keywords:** retinal images, inflection count metric, tortuosity, skeletonization, diagnosis, blood vessels, decision support system, distributed random forest, decision tree

## Abstract

Hypertensive retinopathy severity classification is proportionally related to tortuosity severity grading. No tortuosity severity scale enables a computer-aided system to classify the tortuosity severity of a retinal image. This work aimed to introduce a machine learning model that can identify the severity of a retinal image automatically and hence contribute to developing a hypertensive retinopathy or diabetic retinopathy automated grading system. First, the tortuosity is quantified using fourteen tortuosity measurement formulas for the retinal images of the AV-Classification dataset to create the tortuosity feature set. Secondly, a manual labeling is performed and reviewed by two ophthalmologists to construct a tortuosity severity ground truth grading for each image in the AV classification dataset. Finally, the feature set is used to train and validate the machine learning models (J48 decision tree, ensemble rotation forest, and distributed random forest). The best performance learned model is used as the tortuosity severity classifier to identify the tortuosity severity (normal, mild, moderate, and severe) for any given retinal image. The distributed random forest model has reported the highest accuracy (99.4%) compared to the J48 Decision tree model and the rotation forest model with minimal least root mean square error (0.0000192) and the least mean average error (0.0000182). The proposed tortuosity severity grading matched the ophthalmologist’s judgment. Moreover, detecting the tortuosity severity of the retinal vessels’, optimizing vessel segmentation, the vessel segment extraction, and the created feature set have increased the accuracy of the automatic tortuosity severity detection model.

## 1. Introduction

Fundus images of the retina are an important window for diagnosing several eye diseases, where the blood vessels can be seen clearly on the retina’s surface. It clearly shows as well that the vessel morphology changes. Its variations are tangible signs to identify the severity level of several eye diseases. One such morphological change is vessel tortuosity, the occurrence of turns and twists in the vessel shape [1]. As the twistedness increases, it indicates an increase in eye disease severity, e.g., central retinal vein occlusion (CRVO) [2], diabetic retinopathy [3,4], hypertensive retinopathy [5], systemic hypertension [6], plus disease and retinopathy of prematurity (ROP) [7,8]. Except for hypertensive retinopathy [5], which has few studies to diagnose it, those disorders are thoroughly researched in the literature. Arteriovenous ratio (AVR) alterations [9] and morphological changes in vascular tortuosity [10] are two characteristics of hypertensive retinopathy that are expressed in the retinal wall. AVR is used to determine the severity of hypertensive retinopathy in the majority of currently conducted investigations [11]. Others have added OD detection [11,12], and others added the tortuosity [13]. However, extra efforts are needed in disease diagnosing research. Targeting working on a computer-automated diagnostic system to quantify tortuosity is a challenging task. Although there are several metrics in the literature to quantify tortuosity, each of these metrics has advantages and disadvantages, and more research is required to determine which metrics are the most accurate as well as to standardize the severity levels of tortuosity and correlate these severity levels with each eye disease [14].

In this work, the authors propose a method for auto-detection and identification of the tortuosity severity. Which can be an add-on to the automated system for clinical decision support.

The authors selected fourteen tortuosity metrics and achieved the following results:

- Performed the vessel segmentation of each image AV-classification dataset [15] using the author’s previous work [16] and extracted the vessel segments.
- Calculated the tortuosity values of each of the fourteen tortuosity metrics to the vessels of each image in the large-scale AV-classification dataset [15].
- The result is two tortuosity feature sets, one at the image level and the other at each vessel-segment level.
- Collaborated with two ophthalmologists from RAK university of science and technology and Saqr hospital to label AV-classification dataset images to 4-levels of severity.
- The two ophthalmologists label the images of the AV classification dataset to classify each image to its tortuosity severity level (normal, mild, medium, and severe) based on their expert judgment as ground truth labels for tortuosity severity.
- The feature sets and the expert labels are used as input to machine learning to classify each retinal image into a class from (0 to 4) that tags each image with the severity of retinal vessels tortuosity, whether it is normal, mild, medium, or severe.
- The AV-classification dataset is extended by adding the 4-severity grades of each image.
- Finally, the new extended AV-classification comprehensive dataset is renamed to the retinal vessel morphometry (RVM) dataset. As it is a sizable data set and contains ground truth labels for each of these issues, the data set is made available for use by other researchers to study the tortuosity measures and other categories of retinal fundus image research problems, such as vessel segmentation, artery vein classification, and tortuosity severity.

The rest of this paper has five major sections. Section 2 elaborate on the tortuosity literature review, and Section 3 covers the materials used and the proposed methodology, elaborating on the tortuosity metrics formulas and the proposed method for tortuosity ground-truth creation. Section 4 experimental results and discussion, the proposed tortuosity severity levels, and the updated data set. Finally, Section 6 is the conclusion.

## 2. Retinal Tortuosity Review

Tortuosity metrics have been surveyed and reviewed in several studies. For instance, Abdalla et al. [10] and Abbassi et al. [17] have classified the tortuosity metrics surveyed as distance-based, curvature-based and mixed methods, with a detailed explanation of each specific method and its formula, in addition to reviewing and classifying the tortuosity datasets used in those studies. Kalitzeos et al. [18] has extensively reviewed the tortuosity measures and their clinical applications. Zaki et al. [14] has presented a detailed discussion about diabetic retinopathy’s correlation with vessel tortuosity. Lotmar et al. [19] measured a vessel segment’s arc to chord ratio to determine the length increase between two vessel points. This technique had the limitation of not being sensitive to the segment morphology.

Capowski et al. [20] also implemented the same approach on a selected range of vessel lengths, Heneghan et al. [21], and Swanson et al. [22] improved this technique using a weighted scheme and applied it to ROP cases.

Gelman et al. [22] applied this approach to specially selected images related to ROP cases, and Grisman et al. [23] improved the arc to chord measure by taking into consideration the amplitude of the curve and number of turns. Nidhal Khdhair El Abbadi and Enas Hamood Al Saadi [24] also used the arc to chord measure for the segmentation of vasculature. They used a mask filter over the length of a BV branch to follow each segment of the retina’s blood vessels. Hand-drawn lines only confirmed their algorithm as an alternative to real retinal images.

Wallace et al. [25] used the ROP tool on several points along the vessel. The tortuosity results are from the ratio of the curved length of the segment to the length of the smoothed curve between these points. This approach is user-dependent and is not sensitive to the number of turns in the vessel. Patwari et al. [26] used an image processing technique to extract blood vessels on a DR case to determine the extracted vessel length over distance. It must also be noted that arch-to-cord-based tortuosity measurement techniques are not sensitive to segment morphology, and therefore, their results lack accuracy.

Curvature-based tortuosity measure techniques were introduced in Chandrinos et al.’s [27] work, where the direction change in a segment is introduced by evaluating the local mean angle change method. The technique’s drawback is that the vessel’s branches, with no difference in their course, will not affect the tortuosity measure. Hart et al. [28] calculated the total curvature using integrals; this method is not sensitive to vessel-segment-curve convexity changes. Dougherty and Varro [29] used the coordinates of the vessel-segment midpoint by summing the second derivatives of their coordinates; the accuracy of this method depends on the skeleton extraction phase of the vessel mid-line. Lorga and Dougherty [30] defined tortuosity as the accumulated change of angles along the vessel length and applied it to a type 1 diabetic case.

Wong et al. [3] applied Hart’s integral of total square curvature used in Type 1 diabetes. Faraz Oloumi et al. [31] proposed a novel angle-variation-based tortuosity measure created on Gabor filters to sense vasculature and methods for image processing to skeletonize the image vasculature. Sylvie et al. [32] computed the individual segment tortuosity using curvature parameters such as standard curvature deviation and average curvature value by unit length.

The hybrid tortuosity measures can be summarized in the following works. Mayrhofer-Reinhartshuber. Ref. [33] introduced a multiscale analysis tortuosity detection using wavelet and fractal metrics. Dougherty and Johnson [34] approximated the vessel by a polynomial spline fitting. This approach’s accuracy depends on the data ball size. Danu et al. [35] and Rashmi Turior et al. [36] used a chain code algorithm and applied it using a robust matrix created on the curvature to calculate the blood vessel’s tortuosity level. This approach requires the correct determination of the curvature k-value. Chakravarty and Sivaswamy [37] used a Quadratic Polynomial Decomposition for creating a numerical tortuosity index. The technique can differentiate the size, relative shape, and orientation of the BV bend. However, the suggested algorithm yields less accuracy than Wilson et al. [38]. Hamid Reza Pourreza, Mariam Pourreza, and Touka Banee [39] calculated the local and global tortuosity by applying a circular mask on each skeleton point of the retina vessels. However, the suggested algorithm results have a lesser accuracy than Wilson et al.’s [38] method.

Narasimhan and Vijayarekha [40] introduced a novel approach for determining global tortuosity in clinical perception using machine learning algorithms. After pre-processing and feature extraction, the eight-dimensional feature vector was created by calculating the tortuosity. Then, the top four features were selected by applying the feature selection process depending on the correlation for classification. The performance of this approach was evaluated by utilizing the images collected from the database. By using the SVM classifier, this approach offered the highest sensitivity. Moreover, the overall sensitivity was improved with the feature selection process and reduced computational complexity. With this novel combination of feature-classifier, increased sensitivity was obtained.

Mayrhofer-Reinhartshuber et al. [33] proposed a novel algorithm for automatically categorizing tortuosity in images created from a database named RET-TORT. This algorithm could be implemented depending upon the integration of multiscale wavelet and nonlinear derived analysis, which could be applied directly to the segmented vessel images without suffering from the effects of defective mathematical abstraction or sampling rates that were poorly selected. This helped to enhance reproducibility, and it had the main benefit of identifying tortuosity and decision-making. Moreover, this algorithm was robust against the noise, and it offers better results for venules and arterioles equally.

Mapayi et al. [41] offered research on integrating the difference image and K-means clustering for segmenting the vasculature. In the middle lines of the vessel, the stationary points were utilized for modeling the identification of twists in the vessel branches. In addition, the tortuosity index could be measured by using the integration of the arc-chord along with the stationary points. The experimental results showed that k-means, combined with the different images, robustly accomplished retinal vessel segmentation. The STARE and DRIVE datasets were used to analyze this method’s performance, providing maximum accuracy and sensitivity. This method achieved higher mean accuracy and mean sensitivity with better specificity than other approaches. Additionally, this method attained a strong correlation in the non-normalized tortuosity index, which integrated the distance measure as well as a vessel twist frequency.

Khansari et al. [42] presented a study about the quantitative Vessel Tortuosity Index (VTI) depending on a mixture of local and global structures of the vessels’ center line. This VTI could be applied in the retinal vessels, which were imaged by utilizing the optical coherence tomography angiography in the regions centered on the fovea, such as parafoveal and perifoveal regions. This application of VTI in retinal vessels could be achieved by developing image processing pipelining. Here, the relationship between age and VTI was inspected in the perifoveal areas. This VTI could be calculated from the OCTA images, and they were compared among the NC and SCR by utilizing generalized least square regression. A significant association was determined between the VTI and age in the perifoveal region. The results showed that the VTI was increased in SCR compared to the NC in the parafoveal and perifoveal regions. Moreover, the results depicted that this tested technique offered better results in identifying increased tortuosity in the vessels.

## 3. Materials and Method

This section describes all the materials and methods used in this study to achieve the tortuosity severity labels.

### 3.1. Materials

**Figure 1:**
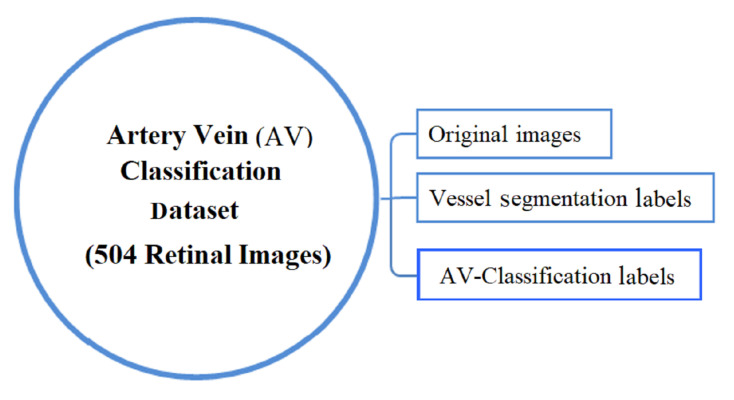
The AV-classification dataset used as input to this research work.

In this work, we have used the AV classification dataset created in [43]. The dataset is ideally suited for supervised deep learning. It contains 504 images with two labels for each in the dataset, the AV classification label and the vessel segmentation label. As illustrated in Figure 1. The fundus images were taken using non-mydriatic fundus cameras (Topcon) from 50 patients in the middle-aged category. Images of the left and right eyes are both available.

Five hundred four labels are developed for each label type (Vessel segmentation label, AV-classification label). The colored vessel segmentation labels are used to run the deep-learning optimized algorithm on the original retinal picture from the AV classification dataset to segment the vessels. The graphics and labels for type-1 and type-2 are 2002×2000 pixels in size. For vascular segmentation and AV classification studies, each original retinal image comprises two labels, one monochrome and the other colored.

At the end of this study, the AV-classification dataset will be extended with labels for each image tortuosity severity (normal, mild, medium, and severe) for each of the 504 images.

### 3.2. Method

**Figure 2:**
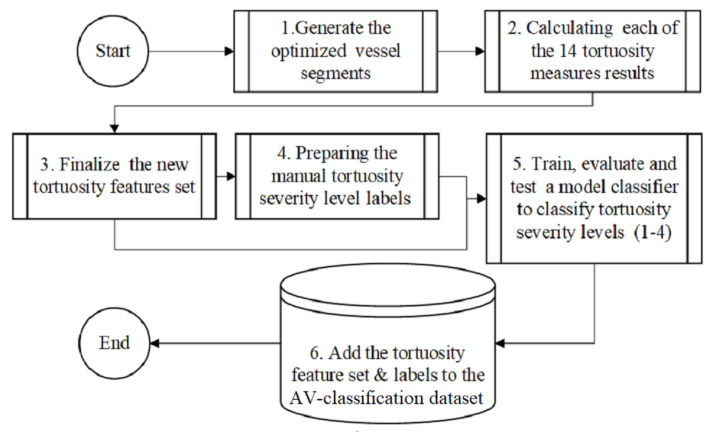
The process of measuring the tortuosity severity levels.

**Figure 3:**
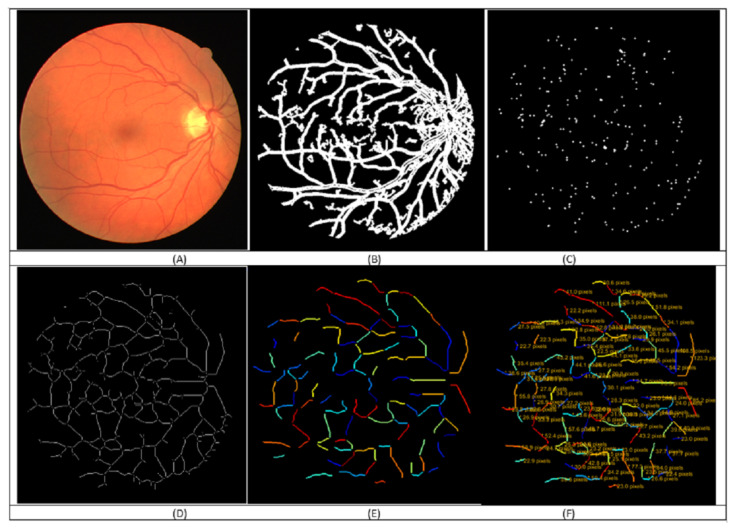
Illustration of the tortuosity calculation steps: (**A**) the fundus image, (**B**) vessels extraction, (**C**) intersection points identification, (**D**) skeletonization, (**E**) vessel segments of fragments segmentation, (**F**) tortuosity calculation for the fourteen metrics.

The retinal touristy severity levels detection in the retinal fundus image method is summarized in Figure 2. The method presents the workflow steps with the retinal images segmented, skeletonized, and devised into segments (see Figure 3). The segments then become ready for applying the fourteen tortuosity metrics. The metrics are calculated and registered for every vessel fragment in the feature-set to classify each retinal image into one of the four severities using machine learning. The tortuosity classification results are checked and confirmed by two ophthalmologists from RAK University for health and medical sciences. Hence, the resulting tortuosity levels are added as 4 Tortuosity severity levels to our AV-classification dataset. The segmentation of the vessels from the colored retinal image is a challenging task. The challenges in this stage include uneven illumination, poor contrast, center light reflex, background artifacts, and choroidal vascularization like impulse noises and background homogenization. A pre-processing course of action increases the discrimination between vessels and the background color by applying morphological processing and normalization. For vessel segmentation, we implemented the method in [44] for effective vessel segmentation, along with the optimization defined in [16].

In the next step, we detect the branch points. Detecting the vessel tree branch points is required to measure, analyze, and quantify the tortuosity level. Branch points are detected by a morphological operation that detects the branch points, dilates them, and reserves the identified branch points pixels that separate different segments [45]. In contrast, the edges of the vessel tree vasculature are obtained through a morphological operation that cleans the inner pixels and retains the pixels on the vessel [45]. After that, vessel skeletonization is approached. In this work, we have used the optimized vessel fragments extraction detailed in [15], where we proposed an enhancement to the skeletonization results by smoothing and removing spur dots from the skeleton iteratively. In addition, a newly introduced technique removes the fake ‘L’-shaped (junction) segment parts. That results in an improvement in the generated segments as eleven vessel branch segments extraction from the vasculature tree. For more details, consult the optimizing vessel fragment extraction in [15], which ends with the optimized vessel segments that are ready for further segment-wise and image-wise morphometric analysis.

### 3.3. Tortuosity Metrics

**Figure 4:**
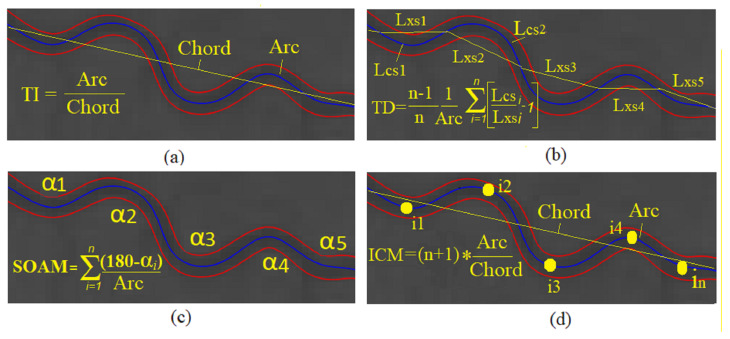
Illustration for arc and chord length in: (**a**) tortuosity index (TI), (**b**) tortuosity density, (**c**) sum of angles metric (SOAM), and (**d**) Inflection count metric (ICM).

Each image’s skeleton extracted segments are traversed. For each vessel segment, the features extraction process starts by calculating the straight line distance and the geodesic distance. This was followed by calculating all fourteen tortuosity measures and creating a record for the segment in the segment level feature set designed to capture the tortuosity attributes for each vessel segment. Finally, the statistical summaries are calculated for each image’s segments that write a row in the image-level-tortuosity feature set file. Figure 4 illustrates sample metrics to measure the vessel tortuosity, whether distance-based measures, curvature-based methods, or others.

All fourteen tortuosity metrics and their required attributes to calculate the tortuosity are defined and explained below:

- Straight Line Distance (*Chord*): It is the straight-line-distance between two endpoints of the centrelines segment skeleton (Euclidian distance).
  (1) $$Chord=\sqrt{{\left(x_{2}-x_{1}\right)}^{2}+{\left(y_{2}-y_{1}\right)}^{2}}$$
- Geodesic distance (*Arc*): The arc-length distance between the segment end- points, using the maximum non-infinity quasi-Euclidean distance between two endpoints of the segment center-line skeleton.
  (2) $$d\left(\gamma \left(t2\right)-\gamma \left(t1\right)\right)=v*\left|t2-t1\right|$$
- Distance Metric (*DM*): Although, the most straightforward dimensionless tortuosity measure used in the literature is the arc to chord ratio between the start and end points of the center line. It does not distinguish between the curvature of “S” and “C” shaped segments with equivalent arc-length. It is the most commonly used metric in the literature [46].
  (3) $$DF=\frac{Arc}{Chord}$$
- Arc length/chord length: Called the distance factor (*DF*) as well. After dividing the segment into subsegments into chosen sample points (*n*) , (*DF*) is the summation arc to chord ratio of each subsegment for all subsegments.
- Tortuosity density (*TD*): After dividing the segment to subsegments into chosen sample points (*n*). *TD* is the summation of each subsegment’s arc to chord ratio for all sub-segments.
  (4) $$TD=\frac{n-1}{n}\frac{1}{Arc}\sum_{i=1}^{n}\left[\frac{Lcs_{i}}{Lxs_{i}}-1\right]$$
- The curvature at a single point (*t*): For a point t(x(t),y(t)) at vessel segment (s), the curvature at a point *t* is defined as the equation
  (5) $$C\left(t\right)=\frac{x^{\prime }\left(t\right)*y^{\prime \prime }\left(t\right)-y^{\prime }\left(t\right)*x^{\prime \prime }\left(t\right)}{{\left(x^{\prime }{\left(t\right)}^{2}+y^{\prime }{\left(t\right)}^{2}\right)}^{\frac{3}{2}}}.$$
- Tortuosity density (τ1): After dividing the segment into subsegments on chosen sample points (*n*), tortuosity density, for straight line segment.
  (6) τ1=DF−1
- Total curvature (τ2): Is the integration of C(t).
  (7) $$\tau 2=\int_{t_{o}}^{t_{n}}C\left(t\right)dt$$
- Total Squared curvature (τ3): Is the integration of $C{\left(t\right)}^{2}$
  (8) $$\tau 3=\int_{t_{o}}^{t_{n}}C{\left(t\right)}^{2}dt$$
- Total curvature/Arc-length (τ4): Is the integration of C(t)/Arc
  (9) $$\tau 4=\int_{t_{o}}^{t_{n}}\frac{C(t)}{Arc}dt$$
- Total squared curvature/Arc-length (τ5): Is the integration of $\frac{C(t)}{Arc}$
  (10) $$\tau 5=\int_{t_{o}}^{t_{n}}\frac{C{\left(t\right)}^{2}}{Chird}dt$$
- Total curvature/Chord-length (τ6): Is the integration of C(t)/Chordlength
  (11) $$\tau 6=\int_{t_{o}}^{t_{n}}\frac{C(t)}{Chord}dt$$
- Total squared curvature/Chord-length (τ7): Is the integration of C(t)∗C(t)/ Chordlength
  (12) $$\tau 7=\int_{t_{o}}^{t_{n}}\frac{C{\left(t\right)}^{2}}{Chord}dt$$
- Sum of angles metric (*SOAM*): It is used for measuring the strongly coiled vessels, and the *SOAM* is a result of measuring the angle between two vectors formed by each consecutive three-segment point. The normalized summation of all of these angles along the segment is measured by segment length [46]. The units of the *SOAM* measure are (radians/mm). This metric requires that points used from a segment to calculate it are evenly spaced.
  (13) $$SOAM=\sum_{i=1}^{n}\frac{\left(180-\alpha_{i}\right)}{Arc}$$
- Inflection count metric (*ICM*): It was extending the *DM* and is known as the DM times the inflection points count along the segment.

The inflection point is the orientation change the Frenet frame of approximately 180 deg of the binomial and the normal axes of [46]. It has been shown to have a substantial tortuosity classification accuracy in [36]

(14) $$ICM=\left(Inflection\_points+1\right)*\frac{Arc}{Chord}$$

### 3.4. Preparing the New Feature-Set

**Figure 5:**
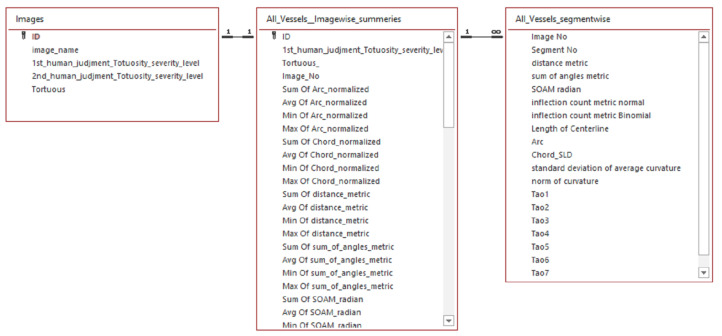
ERD diagram of the image level and segment level feature sets.

The feature set tortuosity calculation is performed for all the AV-classification data set (504) images. Following the procedure in Figure 2, the proposed method starts by segmenting a binary image that contains the segmented vessels of the retina using the optimized method in [16], then using iterative thinning to localize the skeleton of the vessels is extracted. The skeleton is fragmented and optimized to vessel fragment [13], where each vessel fragment connects two intersection/bifurcation or endpoints in the skeleton. Each vessel fragment extracted from the retinal image is considered a curve, and we apply all the fourteen tortuosity mathematical Formulas (1)–(14) listed. The results are to the feature set of all the tortuosity attributes of the vessel fragment. The tortuosity metrics are quantified for each vessel fragment to finalize the fragment-wise features, followed by calculating the summary statistics for each image to finalize the images-level feature-set (see Figure 5).

A procedure of evaluating fourteen tortuosity metrics and adding a newly labeled tortuosity feature-set as an extension to the AV-classification dataset contains the image-wise and vessel segments-wise tortuosity features. In addition, it contains a manual label of tortuosity severity grading and the related fourteen tortuosity measures of the entire images in the AV classification dataset. Two tortuosity feature sets have been introduced. One is segment-level tortuosity features, and the other is image-level statistics tortuosity features. The image-level statistics include the number of segments in the image, and for each tortuosity metric, we quantify the statistical summaries such as (average, minimum, and maximum). An illustration of the ERD diagram of the feature set is in Figure 5.

### 3.5. Tortuosity Labeling Approach

After preparing the features set for each image’s vessel segments, three labels are created to classify each image by an expert ophthalmologist. The first label is to classify the retinal image as tortuous or not (tortuous, not tortuous). The two subsets are achieved by splitting the RVM dataset images into non-tortuous and the rest as tortuous images. The second and third labels are marked to classify the tortuosity severity into four levels of severity (normal, mild, moderate, and severe). The morphological characteristics of the vessels in the retina give us an idea for manually differentiating the severity levels of tortuosity with confidence based on the above tortuosity measures. Finally, a feature set is generated for the 504 images. The tortuosity metrics are derived for every vessel segment, followed by the summary statistics generation for each image. A procedure of evaluating fourteen tortuosity metrics and adding a newly labeled tortuosity feature-set as an extension to the RVM dataset contains the image-wise and vessel segments-wise tortuosity features. In addition, it contains the annotated condition of the images depending on the morphological features and characteristics of the retinal vascular system.

### 3.6. Tortuosity Labeling Methodology

**Figure 6:**
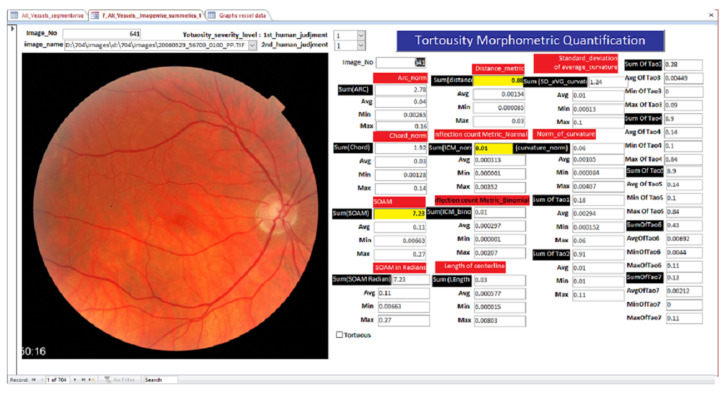
Tortuosity morphometric analysis and quantification form.

The tortuosity severity levels labeling is performed using a custom-developed form illustrated in Figure 6. The form helped attribute each image with the tortuosity level Figure 6 by an ophthalmologist and two computer vision specialists. The labelers use the prepared form to investigate the tortuosity metric values of each retinal image.

This metric reference has been added as another guide for the two ophthalmologists to finalize the manual labeling. Finally, the validation stage consists of a counter validation that involves a computer-vision expert revision and a verification step for each label by the ophthalmologists.

### 3.7. Tortuosity Severity Levels Identification Using Machine Learning

The generated feature set, along with the created labels, is used as input to the three machine learning algorithms J48 decision tree, the ensemble rotation forest, and the distributed random forest, to create four clusters of severity levels from 1 to 4 (normal, mild, moderate, and severe).

The below subsections summarize the machine learning algorithms used in this work.

#### 3.7.1. J48 Decision Tree

A J48 decision tree is a supervised machine learning technique for classification, regression, and knowledge discovery. This DT is an extension to the ID3 algorithm to develop a smaller tree with a newly added generalized option to configure the resampling method to be used in the feature consolidation process. It uses the divide and conquers method to construct the tree for generating a C4.5 decision tree that is pruned or unpruned. J48 is the Consolidated Tree Construction (CTC) method: a collection of sub-samples is used to build a single tree. It calculates entropy and information gain to determine the most useful information for the best tree design.

#### 3.7.2. Rotation Forest

The rotation forest is an ensemble machine learning technique that implements bagging and random sub-spaces. It trains a group of decision trees on a set of randomly chosen data sub-spaces, where each subspace has been transformed using principal components analysis [47].

#### 3.7.3. Distributed Random Forest

A powerful bagging-based ensemble algorithm is the distributed random forest (DRF), which improves learning by addressing the problem of local optima and covering the full search space [48]. It uses a combination of decision trees to maximize the model classification efficiency rather than using just one as a weak learner. Each decision tree in DRF is applied to a subset (bootstrap sample) of the dataset. The individual decision tree is based on the selected random sample and employs an attribute selection indicator for each feature, such as the “Information gain” or “Gini” index.

DRF uses extremely random trees after computing the splits. However, the best of these randomly produced thresholds is chosen as the splitting rule rather than searching for the most discriminative thresholds for each candidate feature. As a result, the model variance can be minimized at the cost of a small bias increase [49]. Finally, each tree votes, and the class with the most popular vote is chosen as the final option. All DT forecasts are combined using a voting procedure to obtain the final product. They give a simple estimate of the conditional distribution. DRF provides a non-parametric estimate of conditional probability P(Y|X=x), which allows for estimating a plethora of studied variables.

**Figure 7:**
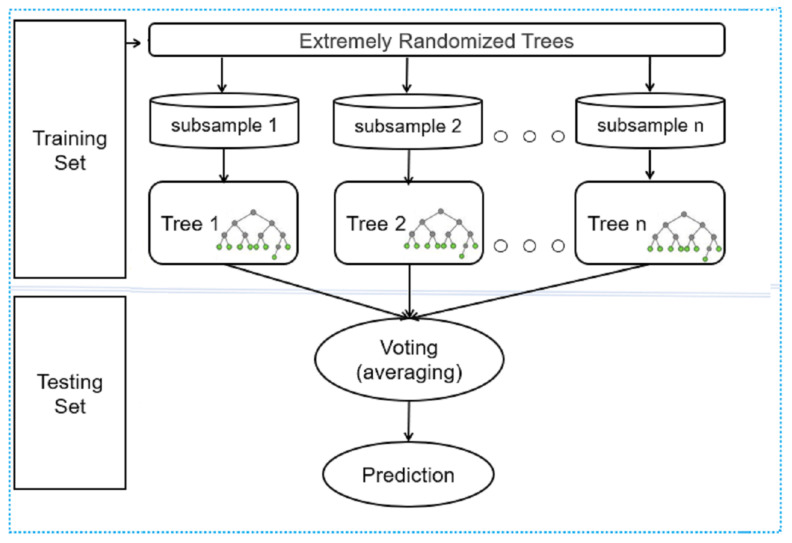
Illustration of the distributed random forest method used with feature set created from the AV-classification dataset.

Furthermore, when compared to other individual machine learning methods, it makes such bagging techniques more robust and accurate (see Figure 7).

The segment-wise feature set in Figure 5 is subdivided into two subsets. One subset is utilized for training, while the other is used for validation and testing of the DRF method. The DRF method generates the final classification model through training, validation, and testing.

The proposed method is developed using the R programming language, and the libraries to reprocess and transform the dataset are dplyr, H2O, and ggplot2 for machine learning and data visualization, respectively.

## 4. Results

The results section includes the following: The results of this work include calculating the fourteen tortuosity metrics and generating the feature set (see sample rows of the segment-wise tortuosity metircs in Table 1) , followed by applying each machine learning experiment (J48 decision tree, rotation forest, and distributed random forest) to the new feature set to learn the proposed tortuosity severity levels. The machine learning experiments are performed on a gaming PC with a core-I7, 16 GB, and 12 GB Ram GTX NVIDIA GPU.

### 4.1. Tortuosity Classification Results

The labels prepared manually for tortuosity in Section 3.6 have empowered the feature set to be used in supervised learning, in addition to the possibility of using it in unsupervised ML methods. Hence, the feature sets are used in several experiments to finalize the grading of tortuosity severity. Three ML methods (J48 DT, rotation forest, and distributed random forest) are applied and achieved the below summary results in each of the three models.

#### 4.1.1. Results of the Tortuosity Grading of Severity Levels Using J48 Decision Tree Model

The first tortuosity grading model is created by training the image level feature-set via the (J48) decision tree model. The 10-fold sampling approach is used in the evaluation and learning stages. The model training time was 0.01 s. Overall, 467 records are correctly classified, which yields a 92.66% accuracy, while 37 records are incorrectly classified, which represents 7.3%, and the Kappa statistic is (0.857). This proposed method achieved 92.66% classification results compared with the human round truth judgment. Furthermore, While classifying the prepared feature set into one of the severity categories, the J48 classifier has demonstrated very good performance (Normal, Mild, Moderate, Severe).

#### 4.1.2. Results of the Tortuosity Grading Using Ensemble Rotation Forest Model

The second experiment has trained and tested rotation-forest for the tortuosity-severity-level classification. The time used to train the model was 215.02 s. As a result, 474 out of 504 retinal images are correctly classified and achieved 91.19% accuracy. The incorrectly classified records are 62, which represents 8.81%. The Kappa statistic obtained is 0.888. Moreover, the MSE was minimized to 0.097.

#### 4.1.3. Results of the Tortuosity Grading Using Distributed Random Forest Model

The second experiment trained and tested the distributed random forest for the tortuosity-severity-level classification. The time used to train the model was 275.19 s. The model has achieved 99.42% accuracy. Moreover, the MSE was reduced to 0.00000182, and the final RMSE was 0.00000194. The final RMSE is 0.18. The rest of the loss measures results are summarized in Table 2.

### 4.2. From ‘AV Classification’ to RVM Data Set

**Figure 8:**
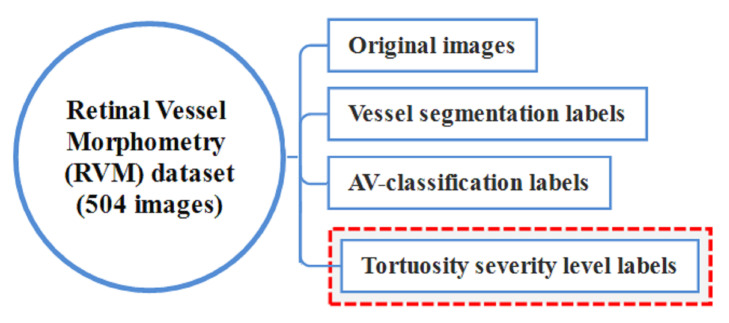
The updated RVM Dataset.

As a final result of this work, the AV classification dataset has been improved by adding tortuosity severity level labels. The updated dataset was renamed to retinal vessel morphometry (RVM) dataset in Figure 8.

The RVM dataset will be available by emailing the corresponding author or at the URLs http://vision.seecs.edu.pk/dlav, accessed on 11 September 2022 or https://docs.google.com/a/seecs.edu.pk/uc?id=1LJf-s4C6zwGwbCUrPy1mG1waqdmx5R-r&export=download&authuser=2, accessed on 11 September 2022. The authors appreciate the feedback of the researchers about using the data set.

#### Visualization of the Tortuosity Classification Results

**Figure 9:**
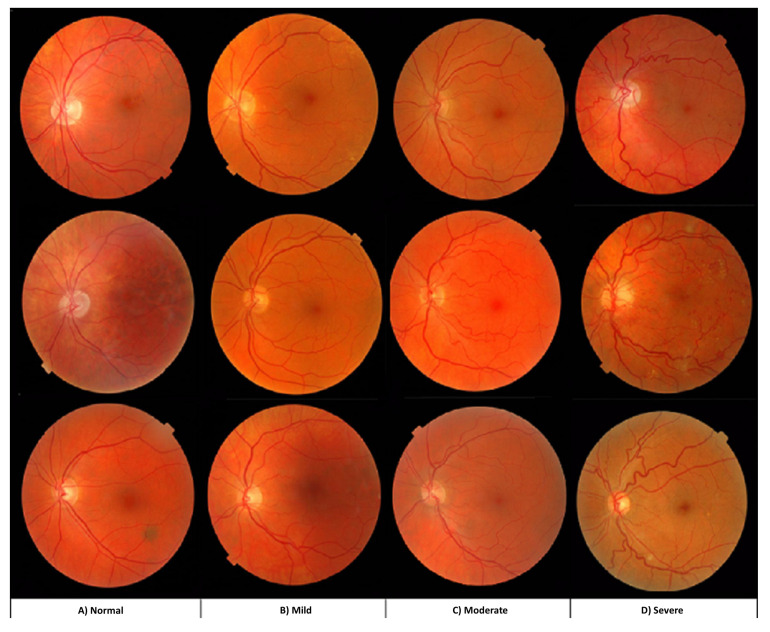
A retinal images sample at every tortuosity grade: (**A**) normal, (**B**) mild, (**C**) moderate, and (**D**) severe.

Figure 9 is a sample retinal image of the tortuosity severity classification. The increase in the twistedness can be visualized with the increased tortuosity severity.

## 5. Discussion

In this work, the tortuosity quantification metrics are reviewed and compared, and fourteen tortuosity metrics are calculated on 504 images of the AV classification dataset. The results created an image-level feature set and a detailed feature set where each row is the detailed calculated tortuosity of each vessel segment. A manual approach is performed to label each image in the dataset that identifies the image’s tortuosity severity level between 1 and 4. The labels are verified and reviewed by a couple of ophthalmologists from Saqr hospital and RAK university for medical and health sciences. The prepared features set has been used in two supervised machine learning methods to identify the tortuosity grading (1–4) of the fundus images in the AV classification database. Finally, we finalized the classification model for grading tortuosity using the decision tree classifier. This classification model has achieved 94.03% accuracy.

The distributed random forest has reported only minimal loss, especially with the 105 trees model. For a detailed discussion, see the Section 5.2. Finally, The J48 decision trees model achieved better tortuosity servility identification results compared to the human labels as it achieved 92.66% compared to the Rotation forest, which achieved only 91.19% of the model accuracy.

### 5.1. The Increase in the Number of Trees Impact

In this study, the implemented DRF algorithm is initially used with 50 trees. In addition, a seed value was used as a constant to guarantee reproducibility. Another model is created using the DRF with a total of 105 trees. These two experiments’ performances are listed in Table 2.

**Figure 10:**
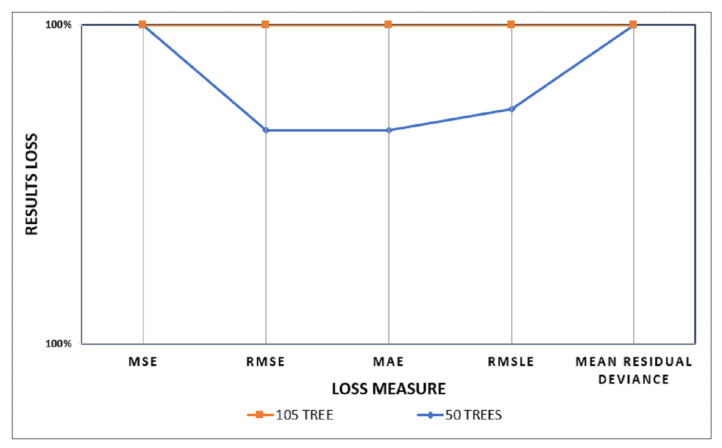
A comparison graph of the loss reported from using the DRF model of 50 Trees versus a 105 trees DRF model loss, in terms of (MSE, RMSE, MAE, RMSLE, and mean residual deviance) loss measures.

The validation results of the two DRF-generated machine learning models indicate that a DRF with a higher tree count will increase the computational cost and considerably reduce the loss. Hence, that will highly improve the results performance. For example, Figure 10 compares both models, where it is clear that the 105 DRF model shows superiority compared with the 50 trees model. That is because the performance of the 100 trees model is closer to 100%, and the five loss measures (MAE, RMSLE, MSE, RMSE, and the mean residual deviance) are all converging to zero, compared with the results of the 50 trees model that clearly show a higher loss.

**Figure 11:**
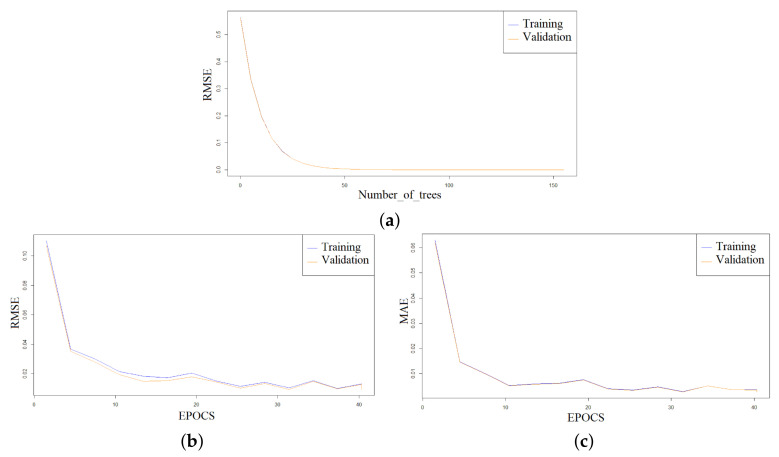
The model training and validation scoring results of RMSE (**a**) vs. number of trees (**b**) vs. number of EPOCS (**c**) and MAE vs. number of EPOCS.

By varying the number of trees giving the training stage and validation stage from 50 to 105 in setting the “number of trees” hyperparameter, the classification errors (MAE, RMSLE, MSE, RMSE, and mean residual deviance) have drastically decreased, hence 105 trees were used to build the final model. Figure 11a presents how the RMSE curve converges asymptotically towards 0.00000194 with the increased number of trees till it reaches 105 trees. It can be seen that the quality of the final model is very high as the validation curve is extremely close to the training curve. In the training and validation phases, the model RMSE is falling towards 0.00000194, which is approximately zero. Additionally, when comparing the number of trees in Figure 11b,c in the two models, we see that the 50 trees model’s RMSE and MAE loss measures converge to 0.00293811 and 0.00276098, respectively. While the 105 trees model, in the training and validation, converges the RMSE and MAE to 0.00000194 and 0.00000182 in both scenarios. The conclusion is that model with 105 trees is more optimal. The four parameters stopping_rounds, stopping_metric, number_of_trees, and stopping_tolerance, which are given the values 3, RMSE, 500, and 0.0005, respectively, affect the optimization behavior.

The hyperparameters suggest that the optimization can be halted if the stopping_metric > 0.0005, and as a result, the RMSE increases while the model was being built, and it was perfectly optimized. However, training and validation break machine learning at 105 trees rather than the full 500 trees when the early stopping logic is applied. Additionally, the model’s performance is significantly impacted by tree numbers from 50 to 105.

### 5.2. Comparison with the Other Methods

In this work, three machine learning models (J48 decision tree, ensemble rotation forest, and distributed random forest) have been performed on the two created feature sets. J48 decision tree model has shown a higher performance than the rotation forest (see Table 3) in terms of F-score, true positive rate (TPR), ROC, Precision (PR), sensitivity (Se), and false-positive rate(FPR). In addition, the rotation forest has shown a very low F-score. On the other hand, this classifier’s disadvantage is that it is slow when dealing with noisy and large datasets. Moreover, it needs high space resources for the repeated use of arrays. In addition, the runtime complexity matches the tree size that cannot be greater than the number of features. Hence, its size grows linearly with the increased quantity of cases [50].

Looking at Table 4 critically, we notice that the model does not learn the normal class properly as it classifies 20 of the normal cases as mild and three severe cases as mild. It is recommended to have more cases of severity 4 to diagnose those models better to avoid over-fitting. Such points suggest further future research work to overcome such issues.

While in the third experiment, the distributed random forest was applied on a combined image-level feature set joined with the vessel-segment level feature set and submitted to the DRF model for learning. As a result, an improved model reports a very small RMSE and MAE, where each converges to zero, and found that the DRF model is more efficient than the rotation forest model and J48 DT model, as is clear in Table 5.

## 6. Conclusions

This paper presents a novel advancement in automated detection and grading of tortuosity severity, which can potentially be used as a clinical decision support system. Image-level tortuosity severity labels that classify the tortuosity of each image as either normal, mild, moderate, or severe were prepared and reviewed by two ophthalmologists from RAK university for medical and health sciences and added as an extension to the previously published AV-classification dataset for all 504 images. The newly extended dataset is named the RVM dataset. Furthermore, in coordination with computer vision experts and ophthalmologists, the manually graded four severity levels were made available to researchers for future studies of tortuosity phenomena. Three classifiers were used to classify the tortuosity severity of the dataset images, J48 decision trees, rotation forest, and distributed random forest, which showed a 92.66%, 91.19%, and 99.42% accuracy in the classification of tortuosity severity of the retinal images, respectively. In addition, the distributed random forest has shown the best results and the least loss in the classification results. Therefore, the distributed random forest-created model was the selected model to be a part of the proposed method to classify the tortuosity severity of any colored fundus retinal image into the (0 to 4) tortuosity severity grades. However, this work can be improved by adding further ophthalmologist human judgment and studying the optimal agreement between them. Furthermore, adding additional images to the dataset, especially for severity 4, will help improve the model, as few cases are available in the current dataset.

## Figures and Tables

**Table 1:** Sample segment-wise tortuosity metrics results.

Image	Seg.	1-A	Arc	Chord	DM	SOAM	ICMn	ICMb	SDavc	Navc	τ1	τ2	τ3	τ4	τ5	τ6	τ7
No.	No.	2-V															
2	42	2	0.12	0.09	0.0001	0.13	0.0007	0.0008	0.01	0.0014	0.0006	0.01	0.0003	0.12	0.1	0.005	0
2	44	2	0.18	0.14	0.0002	0.36	0.0011	0.0009	0.01	0.0022	0.0011	0.01	0.0001	0.12	0.1	0.005	0
2	46	2	0.06	0.04	0.0002	0.13	0.0003	0.0002	0.01	0.0009	0.0014	0.08	0.05	0.25	0.3	0.009	0.004
3	2	2	0.08	0.05	0.0002	0.19	0.0002	0.0003	0.02	0.0015	0.0011	0.01	0.0001	0.12	0.1	0.005	0
3	4	2	0.32	0.27	0.0009	0.13	0.0024	0.0024	0.1	0.0041	0.002	0.14	0.14	0.17	0.2	0.006	0.002
3	5	1	0.02	0.01	0.0002	0.08	0.0001	0.0002	0.01	0.0004	0.0009	0.01	0.0001	0.12	0.1	0.005	0
44	117	1	0.2	0.16	0.0012	0.08	0.0008	0.0012	0.01	0.0021	0.0017	0.26	0.26	0.28	0.3	0.01	0.005
44	119	1	0.003	0.0073	0.03	0.01	0.0011	0.0005	0.0059	0.0001	0.07	0.07	0.06	0.53	0.5	0.06	0.06
45	1	1	0.1	0.07	0.0001	0.13	0.0006	0.0006	0.01	0.0014	0.0009	0.01	0.0003	0.12	0.1	0.005	0.004
45	2	2	0.0078	0.0076	0.02	0.02	0.002	0.0008	0.14	0.0017	0.02	0.04	0.02	0.32	0.3	0.01	0.01
45	3	1	0.02	0.01	0.0011	0.25	0.0005	0.0004	0.03	0.0011	0.0015	0.01	0.0004	0.13	0.1	0.005	0
45	4	2	0.02	0.0079	0.0002	0.05	0	0	0.02	0.0004	0.0012	0.01	0	0.11	0.1	0.005	0

Note: 1-A: Artery, 2-V: Vein, ICMb: Inflection count metric Binomial, ICMn: Inflection count metric normalized, SDavc: Standard deviation of average curvature, Navc: Norm of average curvature.

**Table 2:** Distributed Random Forest two experiments’ results.

Loss Function	50 Trees	105 Trees
MSE	0.00000863	$3.75\times 10^{-12}$
RMSE	0.00293811	$1.94\times 10^{-6}$
MAE	0.00276098	$1.82\times 10^{-6}$
RMSLE	0.00097426	$5.15\times 10^{-7}$
Mean-Residual-Deviance	0.00000863	$3.75\times 10^{-12}$

**Table 3:** Results comparison for the classifiers (J48, ensemble rotation forest).

Model	TPR	FPR	Pr	Se	Fscore	ROC
Decision Tree (J48)	0.927	0.084	0.879	0.927	0.988	0.994
Rotation Forest	0.869	0.132	0.667	0.869	0.484	0.953

**Table 4:** Confusion matrix of the (J48) DT model.

Predicted as →	Normal	Mild	Moderate	Severe
Actual ↓				
**Normal**	2	20	0	0
**Mild**	0	269	0	0
**Moderate**	0	14	196	0
**Severe**	0	3	0	0

**Table 5:** Comparing the loss in distributed random forest with the loss in the J48 decision tree and rotation forest’ results.

Loss Function	J48 Decision Tree	Rotation Forest	DRF 105 Trees Model
RMSE	0.05	0.18	0.00000194
MAE	0.01	0.01	0.00000182

## Data Availability

An email requesting the dataset generated from the corresponding author.

## Abbreviations

A list of abbreviations used in this article:

CRVO	Central retinal vein occlusion
ROP	Retinopathy of prematurity
AVR	Arteriovenous ratio
RVM	Retinal vessel morphometry
VTI	Vessel tortuosity index
TI	Tortuosity index
TD	Tortuosity density
SOAM	Sum of angles metric
ICM	Inflection count metric
DM	Distance metric
DF	Distance factor
RM	Resampling method
CTC	Consolidated tree construction
DRF	Distributed random forest
RMSE	Root-mean-square error
MSE	Mean square error
MAE	Mean absolute error
RMSLE	Root mean squared logarithmic error
Se	Sensitivity
PR	Precision
TPR	True positive rate
FPR	False positive rate
RAKMHSU	RAK Medical and Health Sciences University
